# Association between urinary iodine concentration and the risk of papillary thyroid cancer by sex and age: a case–control study

**DOI:** 10.1038/s41598-023-29071-4

**Published:** 2023-02-04

**Authors:** Yerin Hwang, Hyun-Kyung Oh, Jae Hoon Chung, Sun Wook Kim, Jung-Han Kim, Jee Soo Kim, Myung-Hee Shin

**Affiliations:** 1 Department of Social and Preventive Medicine, Sungkyunkwan University School of Medicine, Suwon-si, South Korea; 2 Department of Benefits Innovation, National Health Insurance Service, Wonju-si, South Korea; 3 Division of Endocrinology and Metabolism, Department of Medicine, Thyroid Center, Samsung Medical Center, Sungkyunkwan University School of Medicine, Suwon-si, South Korea; 4 Department of Surgery, Samsung Medical Center, Sungkyunkwan University School of Medicine, Suwon-si, South Korea

**Keywords:** Diseases, Risk factors

## Abstract

Previous studies on dietary iodine intake and the risk of papillary thyroid cancer (PTC) have demonstrated inconsistent results. We aimed to evaluate the association between urinary iodine concentration (UIC), a surrogate biomarker for dietary iodine intake, and the risk of thyroid cancer stratified by sex and age in an iodine-sufficient area. A hospital-based case–control study was conducted in Seoul, South Korea. A total of 492 cases of newly diagnosed PTC and 595 controls were included. Compared with the lowest quartile of creatine-adjusted UIC (< 159.3 μg/gCr), the highest quartile (≥ 1037.3 μg/gCr) showed an increased risk of PTC (odds ratio [OR] 1.49, 95% confidence interval [CI] 1.04–2.13), especially in those who were < 45 years old (*p*_trend_ = 0.01) compared with those who were ≥ 45 years old (*p*_trend_ = 0.48). For those who were < 45 years old, a positive association between creatinine-adjusted UIC and the risk of PTC was observed in both men (q4 vs. q1, OR 4.27, 95% CI 1.14–18.08) and women (OR 1.97, 95% CI 1.04–3.78). For those who were ≥ 45 years old, no association was found in any sex. Creatinine-adjusted UIC was positively associated with the risk of PTC, especially in those who were younger than 45 years for both men and women.

## Introduction

The incidence of thyroid cancer has been increasing in several countries, and the age-standardized rate (ASR) of thyroid cancer worldwide in 2020 was estimated to be 6.6 per 100,000 people^1^. In South Korea, thyroid cancer cases accounted for 12% of the total number of cancer cases for both sex combined in 2019, ranking first among the incidence rates of different types of cancer^2^. The incidence rate was 90.0 per 100,000 in women and 29.3 per 100,000 in men. The age-specific thyroid cancer incidence rate peaked in the age range of 40–44 and decreased with a parabola shape for both women and men in South Korea.

The rapid increase in the incidence of thyroid cancer can be attributed to the development of high-resolution ultrasound technology, resulting in an increase in the rate of microcarcinoma diagnosis through ultrasound-induced fine-needle aspiration cell examination^3^. The increase has mostly been accounted for by an overdiagnosis of subclinical lesions^4^. Papillary thyroid cancer (PTC) is increasing in a steeper pattern than are other histological shapes, especially for micropapillary cancer with a tumor size of less than 1 cm^5,6^. The proportion of PTC among all types of thyroid cancer in South Korea increased from 79.7% in 1995 to 95.7% in 2018^7,8^. Some could argue that higher opportunities to obtain thyroid ultrasound screening, as it was frequently included in health examination programs, might account for the high incidence. However, even in the 15- to 19-year-old age group, who rarely undergo thyroid cancer screening, thyroid cancer is the most common type of cancer in both male and female populations in South Korea^2^. Therefore, the steep increase in incidence of thyroid cancer in South Korea cannot be fully accounted for by overdiagnosis or excessive health screening, and factors associated with the real risk increase should be investigated.

An iodine-rich diet is another factor that could account for the high incidence of thyroid cancer in South Korea. The usual Korean diet is very rich in iodine. Additionally, it is customary to eat seaweed soup on birthdays, and women in the postpartum period eat seaweed soup almost every day for months. Both deficient^9–11^ and excess^12,13^ intake of iodine showed an association with an increased risk of thyroid cancer in human populations, with mixed results depending on ethnicity and dietary iodine levels. A study on multiethnic women living in San Francisco Bay in the United States reported that high iodine intake may lower the risk of thyroid cancer^9^. However, in Japanese women with a high iodine intake, those who consumed seaweed daily had an approximately 1.86-times-higher risk of thyroid cancer than those who consumed seaweed less than twice a week^12^.

One of the reasons for the inconsistencies in the association between dietary iodine intake and the risk of thyroid cancer was the difficulty in the accurate assessment of dietary iodine intake due to the incomplete food composition table for iodine and large variation in iodine content by food source. Since over 90% of dietary iodine absorbed in our body is excreted through urine within 1 to 2 days, urinary iodine concentration (UIC) has been used as the standard means to assess the population iodine status^14^. UIC is a less desirable biomarker to assess iodine status at the individual level because it reflects only the recent diet, not the long-term usual diet. However, when accurate dietary iodine assessment is unavailable, UIC could be a good surrogate biomarker for iodine intake.

Several studies have investigated the association between UIC and thyroid diseases^15,16^ and thyroid cancer^17–19^, but to our knowledge, well-designed epidemiologic studies on the association between UIC and the risk of thyroid cancer are sparse.

Therefore, we conducted a hospital-based case–control study to investigate the association between UIC, a surrogate biomarker for iodine intake, and the risk of thyroid cancer in South Korea, where iodine intake is sufficient. The secondary purpose of this study was to investigate possible interaction by sex and age because Korean women have additional exposure to high dietary iodine during the postpartum period, and the age-specific incidence curve of thyroid cancer changes its slope after 45 years of age^20,21^.

## Materials and methods

### Study population

This study included outpatient clinic patients of the Thyroid Center at Samsung Medical Center (SMC), Seoul, South Korea, from November 2011 to June 2016. Research team doctors actively recruited eligible case and control patients. The Division of Endocrinology and Metabolism and Department of Surgery were congregated together at the Thyroid Center, and the research interviewers waited on-site every day and conducted the comprehensive risk factor survey whenever patients were recruited. We planned to recruit cases and controls with 1:1 ratio. However, cases and controls were not individually matched but were frequency-matched for age and sex as closely as possible. All interviews, measurements, and biospecimen collections were conducted in the same manner for the case and control groups.

**Figure 1:**
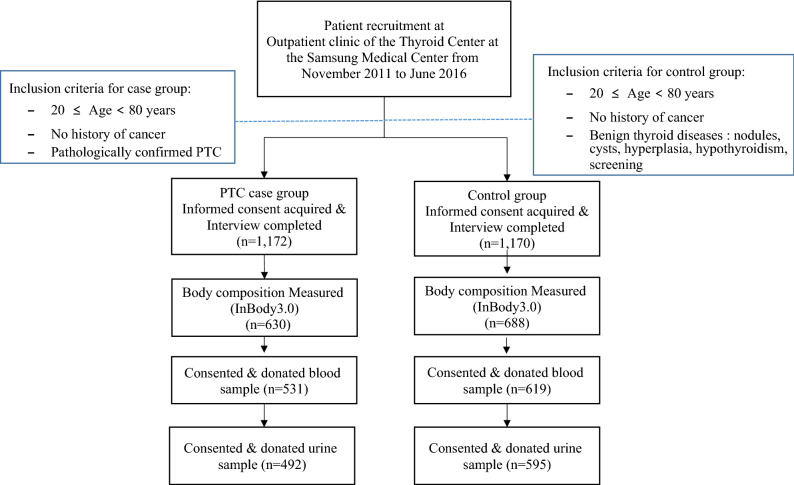
Recruitment of case–control study participants and study flow chart.

The case group included patients aged 20–80, newly diagnosed with pathologically confirmed PTC, with or without follicular variant, who did not have any history of cancer. Those who were found not to have cancer according to the pathologic report after the surgery were excluded. After further excluding those who did not finish the interview (n = 17) and those who were younger than 20 years old (n = 2), a total of 1172 cancer patients completed the interview. Of those, we included in this study 492 cancer patients who actually consented to donate urine samples. There was no significant difference in basic characteristics between urine donors and nondonors in the case group, except for the proportion of men (21.7% among donors, 27.2% among nondonors, p = 0.039) and the proportion of cases with a history of benign thyroid diseases (21.2% among donors, 34.8% among nondonors, p < 0.001). The control group included patients aged 20–80 with benign thyroid diseases, such as thyroid nodules, cysts, hyperplasia, hypothyroidism, etc. or self-induced thyroid screening, who did not have any history of cancer. As healthy as possible, patients were selected as controls, and many of the controls had no plans for long-term follow-up. When a control patient was diagnosed with PTC later in time during the study period, he/she was switched to the case group. After excluding those who did not finish the interview (n = 29) and those who were younger than 20 years old (n = 2), a total of 1170 control patients completed the interview. Of those, 595 controls who actually consented to donate urine samples were included in this study. There was no significant difference in basic characteristics between urine donors and nondonors in the control group, except for the proportion of controls with a history of benign thyroid diseases (34.3% among donors, 44.0% among nondonors, p = 0.001) (Fig. 1).

### Data collection

Research interviewers explained the purpose and contents of the survey to the patients, obtained written consent, and conducted interviews using a structured questionnaire on various risk factors, including lifestyle, medical history, radiation, and diet. For the cancer case group, the interview was conducted before surgery. Weight and height were measured in a shoeless state using an automatic height and weight machine. After the interview was completed, participants were led to the Diabetes Education Center to measure body composition using InBody3.0 (BioSpace). Ten milliliters of whole blood and 12 ml of spot urine samples were collected in accordance with the next diagnostic test schedule in the SMC’s central lab. The fasting time before sample collection was 4 h for afternoon appointments to 12 h for morning appointments. Collected samples were stored at − 20 °C on-site and then delivered every day to the Seegene central laboratory, the collaborating commercial laboratory for our research group, and then aliquoted and stored at − 70 °C.

Questionnaires were doubly entered into the database by two interviewers independently, and the data were compared using Statistical Analysis System (SAS) ver.9.4. When the program identified inconsistencies in the data, we returned to the original questionnaire and made necessary corrections to minimize the input errors.

Body mass index (BMI) was calculated by dividing the measured weight (kg) by the square of the height (m^2^). Smoking status was divided into nonsmokers, past smokers, and current smokers. Those who smoked less than 20 packs of cigarettes in their lifetime were classified as nonsmokers, and those who smoked more than 20 packs were further classified into past smokers and current smokers according to their current smoking status. The daily intake of alcohol was calculated based on the frequency and amount of alcohol intake over 1 year and the types of alcohol consumed, such as makgeolli (Korean rice wine), wine, soju, beer, and spirits. Supplement intake combined the intake frequencies of multivitamins, vitamin C, vitamin E, vitamin D, calcium, omega 3, and red ginseng. Physical activity was classified as yes or no according to whether regular exercise enough to sweat on the body was performed. Education level was classified as elementary school or less, middle school diploma, high school diploma, or college degree or higher. Family history of cancer included cancer in parents and children, and history of thyroid disease included thyroid nodules and benign tumors, hypothyroidism, hyperthyroidism, goiter, and other thyroid diseases.

UIC was measured from a spot urine sample by inductively coupled plasma-mass spectrometry (ICP-MS, Perkin Elmer, ICPMSD, Waltham, MA, USA), the creatinine level was measured by Jaffe (C702, Roche, Mannheim, Germany), and the iodine/creatinine level ratio (μg/gCr) was used as the creatinine-adjusted UIC to minimize diurnal and day-to-day variation^22^. Nevertheless, as mentioned before, UIC had limitations in reflecting long-term usual dietary iodine intake at the individual level. Therefore, rather than using absolute cut-off points, such as 100 μg/L for deficiency, we used the quartiles of the creatinine-adjusted UIC in the controls (< 159.3 μg/gCr, 159.3–394.3 μg/gCr, 394.3–1037.3 μg/gCr, and ≥ 1037.3 μg/gCr) to categorize the participants into four groups.

### Statistical analyses

Categorical variables were expressed as the frequency and percentage, and the chi-square test was used to compare the characteristics of the participants between the case group and control group. Continuous variables are presented as the mean and standard deviation (SD), and a t-test was performed to compare the continuous variables between the two groups.

Age, sex, educational level, physical activity, supplement intake, BMI, daily alcohol intake, smoking status, family history of cancer, and history of benign thyroid diseases were adjusted for as confounding variables. Odds ratios (ORs) and 95% confidence intervals (CIs) of the risk of thyroid cancer were calculated for participants exposed to the higher creatinine-adjusted UICs (μg/gCr) compared with those exposed to the lowest creatinine-adjusted UIC using unconditional logistic regression. Also, we performed the logistic regression using continuous form of the creatinine-adjusted UIC. Since creatinine-adjusted UIC had very skewed distribution, we transformed it to the log of creatinine-adjusted UIC for modeling. To evaluate possible interactions by sex and age, stratified analyses were also performed. *P* for interaction was calculated by adding the product interaction term to the model, and the results are presented under the tables. All analyses were conducted using R, version 3.6.3, and a two-tailed *p* value of < 0.05 was defined as significant.

This study was approved by the institutional review board of Samsung Medical Center (IRB No. 2011-11-025, 2011-11-076). Informed consent was obtained from all subjects and their legal guardians. All methods were performed in accordance with the relevant guidelines and regulations.

## Results

The general characteristics of the participants in the case and control groups are summarized in Table 1. Men comprised 19.9% of the participants. The average age of the participants in the case group was younger (46.5 years) than that in the control group (49.7 years, p < 0.001). The daily intake of alcohol in those in the case group was lower than that of those in the control group, whereas the rate of physical activity and supplement intake were higher, but the differences were not significant. A higher proportion of people had a past history of benign thyroid diseases in the control group than in the case group (p < 0.001). The median value of UIC in the case group was 472.0 μg/gCr and that in the control group was 353.1 μg/gCr.

**Table 1:** Characteristics of the 492 case patients with PTC and 595 control patients with benign thyroid diseases at Samsung Medical Center (SMC) in South Korea between 2011 and 2016.

Characteristics	Total	Case	Control	P-value^a^
N	1087	492	595	
Sex				0.182
Men	216 (19.9)	107 (21.7)	109 (18.3)	
Women	871 (80.1)	385 (78.3)	486 (81.7)	
Age (years)	48.2 ± 10.8	46.5 ± 11.1	49.7 ± 10.3	< 0.001
BMI (kg/m^2^)	24.1 ± 3.3	24.2 ± 3.4	24.1 ± 3.3	0.741
Smoking status				0.869
Never	855 (78.7)	385 (78.3)	470 (79.1)	
Former	148 (13.6)	70 (14.2)	78 (13.1)	
Current	83 (7.6)	37 (7.5)	46 (7.7)	
Daily alcohol intake (g/day)	5.9 ± 16.7	7.0 ± 18.7	5.0 ± 14.7	0.063
Physical activity				0.145
No	629 (57.9)	297 (60.4)	332 (55.8)	
Yes	458 (42.1)	195 (39.6)	263 (44.2)	
Education level				0.440
≤ Elementary school graduation	59 (5.5)	26 (5.3)	33 (5.6)	
Middle school graduation	73 (6.8)	28 (5.7)	45 (7.6)	
High school graduation	384 (35.5)	168 (34.4)	216 (36.5)	
≥ College graduation	565 (52.3)	267 (54.6)	298 (50.3)	
Supplement intake^b^				0.147
No	541 (49.8)	257 (52.3)	284 (47.7)	
Yes	545 (50.2)	234 (47.7)	311 (52.3)	
History of benign thyroid diseases prior to recruitment^c^				< 0.001
No	778 (71.6)	387 (78.8)	391 (65.7)	
Yes	308 (28.4)	104 (21.2)	204 (34.3)	
Family history of cancer				0.694
No	734 (67.6)	335 (68.4)	399 (67.1)	
Yes	351 (32.4)	155 (31.6)	196 (32.9)	
Reasons for visiting outpatient clinic				0.075
Symptom	143 (13.2)	60 (12.2)	83 (14.0)	
Medical examination	903 (83.3)	419 (85.5)	484 (81.5)	
Other department	38 (3.5)	11 (2.2)	27 (4.5)	
Season of the urine collection				0.389
Spring	360 (33.2)	160 (32.6)	200 (33.7)	
Summer	264 (24.3)	121 (24.6)	143 (24.1)	
Fall	189 (17.4)	95 (19.4)	94 (15.8)	
Winter	272 (25.1)	115 (23.4)	157 (26.4)	
Time of the urine collection				0.054
Morning	717 (66.6)	309 (63.6)	408 (69.2)	
Afternoon	359 (33.4)	177 (36.4)	182 (30.9)	
UIC (μg/L), median	385.41	472.00	353.08	
Creatinine-adjusted UIC (μg/g Cr), median	436.45	477.31	394.28	

UIC urinary iodine concentration.

Values are mean ± SD or n (%).

^a^P values were derived from a chi-square test for categorical variables and from t-test for continuous variables.

^b^Supplement intake includes multiple vitamin, vitamin C, vitamin E, vitamin D, calcium, omega-3, red ginseng.

^c^History of benign thyroid diseases prior to recruitment include thyroid nodule or benign tumor, hypothyroidism, hyperthyroidism, goiter, others.

Table 2 exhibits the association between the risk of PTC and continuous form and quartiles of creatinine-adjusted UIC after adjusting for confounders. The highest quartile of creatinine-adjusted UIC (≥ 1037.3 μg/gCr) exhibited a 1.49-fold higher risk of thyroid cancer than the lowest quartile of creatinine-adjusted UIC (< 159.3 μg/gCr).

**Table 2:** The odds ratios (ORs) and 95% confidence intervals (CIs) of the risk of PTC for creatinine-adjusted UIC among 492 case patients with PTC and 595 control patients with benign thyroid diseases at Samsung Medical Center in South Korea between 2011 and 2016.

	Case	Control	OR (95% CI)	OR (95% CI)*	OR (95% CI)**
Continuous log (creatinine-adjusted UIC)	492	595	1.11 (1.05–1.17)	1.11 (1.05–1.17)	1.11 (1.05–1.18)
Quartiles of creatinine-adjusted UIC					
< 159.3 μg/g Cr	107 (21.7)	149 (25.0)	Ref.	Ref.	Ref.
159.3–394.3 μg/g Cr	115 (23.4)	149 (25.0)	1.07 (0.76–1.52)	1.10 (0.78–1.57)	1.17 (0.81–1.69)
394.3–1037.3 μg/g Cr	117 (23.8)	148 (24.9)	1.10 (0.78–1.56)	1.19 (0.83–1.69)	1.14 (0.79–1.66)
≥ 1037.3 μg/g Cr	153 (31.1)	149 (25.0)	1.43 (1.02–2.00)	1.47 (1.04–2.06)	1.49 (1.04–2.13)
p-trend			0.0376	0.0241	0.0371

*PTC* papillary thyroid cancer, *UIC* urinary iodine concentration.

*Adjusted for age and sex.

**Adjusted for age, sex, education level, physical activity, supplement intake, BMI, daily alcohol intake, smoking status, family history of cancer, and history of benign thyroid diseases.

The results of the stratified analysis by sex are presented in Table 3. In women, the highest quartile of the creatinine-adjusted UIC group exhibited a significantly higher risk of thyroid cancer than the lowest quartile of the creatine-adjusted UIC group (OR 1.56, 95% CI 1.04–2.34). There was no significant association between creatinine-adjusted UIC and the risk of thyroid cancer in men (p for trend = 0.3795). Similarly, there was stronger association between continuous log creatinine-adjusted UIC and the risk of thyroid cancer in women (OR 1.12, 95% CI 1.05–1.20) than in men (OR 1.11, 95% CI 0.97–1.29). No significant interaction was observed (*p*_interaction_ = 0.40).

**Table 3:** The odds ratios (ORs) and 95% confidence intervals (CIs) of the risk of PTC for creatinine-adjusted UIC among 492 case patients with PTC and 595 control patients with benign thyroid diseases stratified by sex.

	Men			Women		
	Case	Control	OR* (95% CI)	Case	Control	OR* (95% CI)
Continuous log (creatinine-adjusted UIC)	107	109	1.11 (0.97–1.29)	385	486	1.12 (1.05–1.20)
Quartiles of creatinine-adjusted UIC						
< 159.3 μg/g Cr	27 (25.2)	31 (28.4)	Ref.	80 (20.8)	188 (24.3)	Ref.
159.3–394.3 μg/g Cr	24 (22.4)	22 (20.2)	1.56 (0.66–3.75)	91 (23.6)	127 (26.1)	1.11 (0.74–1.69)
394.3–1037.3 μg/g Cr	25 (23.4)	24 (22.0)	1.52 (0.64–3.70)	92 (23.9)	124 (25.5)	1.13 (0.74–1.71)
≥ 1037.3 μg/g Cr	31 (29.0)	32 (29.4)	1.47 (0.67–3.27)	122 (31.7)	117 (24.1)	1.56 (1.04–2.34)
*p*-trend			0.3795			0.0346

*PTC* papillary thyroid cancer, *UIC* urinary iodine concentration.

*Adjusted for age, education level, physical activity, supplement intake, BMI, daily alcohol intake, smoking status, family history of cancer, and history of benign thyroid diseases.

*p* for interaction = 0.4040.

When we stratified the analysis into those who were < 45 years old and those who were ≥ 45 years old, the association between the creatinine-adjusted UIC and the risk of thyroid cancer appeared in those aged younger than 45 years (q4 vs. q1, OR 2.22, 95% CI 1.27–3.94) but not in those aged 45 or older (q4 vs. q1, OR 1.15, 95% CI 0.72–1.83); however, the interaction was not significant (*p*_interaction_ = 0.11) (Table 4). When we stratified the analysis further by sex, for those who were aged younger than 45 years, creatinine-adjusted UIC was associated with the risk of thyroid cancer in both men (q4 vs. q1, OR 4.27, 95% CI 1.14–18.08) and women (q4 vs. q1, OR 1.97, 95% CI 1.04–3.78). The continuous form of log creatinine-adjusted UIC showed similar age-specific associations with the risk of thyroid cancer.

**Table 4:** The odds ratios (ORs) and 95% confidence intervals (CIs) of the risk of PTC for creatinine-adjusted UIC among 492 case patients with PTC and 595 control patients with benign thyroid diseases stratified by age 45 years old and sex.

Creatinine-adjusted UIC	< 45 years			≥ 45 years		
	Case	Control	OR (95% CI)	Case	Control	OR (95% CI)
Total						
Continuous log (creatinine-adjusted UIC)	209	191	1.17 (1.07–1.29)	283	404	1.08 (1.00–1.17)
Quartiles of creatinine-adjusted UIC						
< 159.3 μg/g Cr	48 (23.0)	63 (33.0)	Ref.	59 (20.8)	86 (21.3)	Ref.
159.3–394.3 μg/g Cr	55 (6.3)	47 (24.6)	1.55 (0.88–2.74)	60 (21.2)	102 (25.2)	0.91 (0.56–1.49)
394.3–1037.3 μg/g Cr	40 (19.1)	39 (20.4)	1.28 (0.69–2.38)	77 (27.2)	109 (27.0)	0.98 (0.61–1.57)
≥ 1037.3 μg/g Cr	66 (31.6)	42 (22.0)	2.22 (1.27–3.94)	87 (30.7)	107 (26.5)	1.15 (0.72–1.83)
* p*-trend			0.0127			0.4787
Men						
Continuous log (creatinine-adjusted UIC)	51	40	1.21 (1.00–1.60)	56	69	1.05 (0.84–1.32)
Quartiles of creatinine-adjusted UIC						
< 159.3 μg/g Cr	12 (23.5)	13 (32.5)	Ref.	15 (26.8)	18 (26.1)	Ref.
159.3–394.3 μg/g Cr	12 (23.5)	9 (22.5)	1.68 (0.43–6.75)	12 (21.4)	13 (18.8)	1.35 (0.41–4.52)
394.3–1037.3 μg/g Cr	11 (21.6)	10 (25.0)	1.97 (0.46–9.08)	14 (25.0)	14 (20.3)	1.76 (0.56–5.72)
≥ 1037.3 μg/g Cr	16 (31.4)	8 (20.0)	4.27 (1.14–18.08)	15 (26.8)	24 (34.8)	0.97 (0.33–2.85)
* p*-trend			0.0410			0.9900
Women						
Continuous log (creatinine-adjusted UIC)	158	151	1.15 (1.05–1.29)	227	335	1.09 (1.00–1.19)
Quartiles of creatinine-adjusted UIC						
< 159.3 μg/g Cr	36 (22.8)	50 (33.1)	Ref.	44 (19.4)	68 (20.3)	Ref.
159.3–394.3 μg/g Cr	43 (27.2)	38 (25.2)	1.54 (0.80–2.96)	48 (21.1)	89 (26.6)	0.84 (0.49–1.46)
394.3–1037.3 μg/g Cr	29 (18.4)	29 (19.2)	1.29 (0.63–2.65)	63 (27.8)	95 (28.4)	0.91 (0.54–1.54)
≥ 1037.3 μg/g Cr	50 (31.6)	34 (22.5)	1.97 (1.04–3.78)	72 (31.7)	83 (24.8)	1.24 (0.74–2.11)
* p*-trend			0.0642			0.3203

*PTC* papillary thyroid cancer, *UIC* urinary iodine concentration.

*Adjusted for education level, physical activity, supplement intake, BMI, daily alcohol intake, smoking status, family history of cancer, and history of benign thyroid diseases.

*p* for interaction in total = 0.1172; in men = 0.2428; in women = 0.2746.

For sensitivity analysis, we changed the age limit for stratification from age 45 to age 50. The result was similar; the association between the creatinine-adjusted UIC and the risk of PTC appeared in those aged younger than 50 years (q4 vs. q1, OR 1.97, 95% CI 1.23–3.16) but not in those aged 50 or older (q4 vs. q1, OR 0.96, 95% CI 0.56–1.66, *p*_interaction_ = 0.07). In further stratification by sex, the association weakened in men (q4 vs. q1, OR 2.42, 95% CI 0.85–7.17) but persisted in women (q4 vs. q1, OR 1.93, 95% CI 1.13–3.33) for those aged younger than 50 years. Using creatinine-unadjusted UIC did not change the result. Compared to those with the lowest quartile of the UIC (< 138.1 μg/L), those with the highest quartile of the UIC (> 936.4 μg/L) had a 1.54 (95% CI 1.05–2.26) times higher risk of PTC.

## Discussion

In this study, we observed the association between urinary iodine concentration (UIC) and PTC risk in younger (< 45 years old) men and women in South Korea, a country well known for its excessive iodine intake and high thyroid cancer incidence. We used the age stratification criteria as 45 years old because the age-specific incidence rate of thyroid cancer in South Korea peaked at age 40–44 and subsequently decreased for both men and women. It is interesting that our result supports the increasing period of the age-related incidence curve.

Urinary iodine excretion is the most widely used biomarker of recent iodine intake, as more than 90% of iodine consumed as food is excreted in urine after being metabolized^23,24^. A 24-h urine test is the gold-standard method, but it has a risk of selection bias due to low compliance^25^. We used a single spot urine test in this study. The spot urine test is easier and has an advantage of being able to test many more participants than the 24-h urine test, but it is highly affected by the urine volume, diurnal iodine changes^22,25^, or even by season^14^. To minimize these variations, the iodine/creatinine ratio was used as the urinary iodine level measure in this study. Nevertheless, the spot urine test cannot replace the 24-h urine test^26,27^. Reduced accuracy due to single spot urine would introduce nondifferential misclassification, and the magnitude of true association might be underestimated.

Since UIC is greatly affected by recent diet, a single UIC may not be a suitable marker for a patient’s long-term dietary iodine intake, especially when the patient has changed his/her diet recently. We collected spot urine on the day of the next outpatient visit, that is, the preoperational visit for cancer patients and the routine follow-up visit for control patients. No dietary guidelines were given to cases and controls at the time of urine collection. All participants were on their usual diet, and it is unlikely that either cases or controls would have changed their iodine intake differentially from their comparison group. Daily fluctuation of dietary iodine intake would also be similar between cases and controls.

The mechanism of carcinogenesis by iodine excess is unclear. One of the potential mechanisms is by modifying thyroid stimulating hormone (TSH), which can promote thyroid cancer. Sudden ingestion of large amounts of iodine inhibits the binding of iodine to Tg via the Wolff–Chaikoff effect^28^ and induces transient elevation of TSH. By chronic intake of excess iodine, however, TSH returns to normal levels in animal studies^13^. In human intervention studies, an excess or safe upper limit of iodine supplementation for more than 4 weeks induced an immediate TSH surge, which remained high after the termination of the intervention^29^. The effect of iodine-excess on TSH in the longer term is not clear. Another possible explanation is that iodine excess can promote a certain genetic type of thyroid cancer. BRAF mutations in PTC were reported to be more frequent in an iodine-excess area than in an iodine-deficient area, although the overall incidence of PTC in the two regions was unknown^13^. Further studies are needed to clarify this relationship at the individual level.

Previous studies examining the relationship between UIC and PTC compared the median values of UIC between the PTC and control groups and reported no significant differences^17,18^. However, a retrospective clinical study conducted in Korea showed a U-shaped association between UIC and the risk of thyroid cancer^19^. Recently, Kim et al. reported with 446 hospital-based PTC cases and 500 community-based controls that those with creatinine-adjusted UIC ≥ 220 μg/gCr had an 18.13 times (95% CI 8.87–37.04) higher risk of PTC than those with UIC, 85 to 219 μg/gCr. The OR of the risk of papillary thyroid microcarcinoma for the same level of UIC was 8.02 (95% CI 4.64–13.87)^30^. The positive association was in line with our study, although the magnitude of their association was much larger than that of our study. There are several possible explanations for this. First, the distribution of creatinine-adjusted UIC among the case group and control group is different from our study. The proportion of UIC > 300 μg/L among the case group was 93.3% in Kim et al.’s study and 59.1% in ours, and the proportion of UIC > 300 μg/L among the control group was 43.4% in Kim et al.’s study and 55.0% in ours. A much wider difference in UIC between the case and control groups existed in Kim et al.’s study. According to the Korean National Health and Nutrition Examination Survey 2013–2015, the national median UIC was 293.9 μg/L^27^. The control group in our study had a slightly higher median UIC (353.1 μg/L). Among 595 controls, 421(70.8%) patients had thyroid nodule. Epidemiologic studies have reported both positive^31^ and inverse^15,32^ associations between iodine excess and the risk of thyroid nodules, and one Korean study reported higher iodine intake in patients with thyroid nodules than in general population^33^. If we assume our control group had a higher intake of iodine than general population, we might have underestimated the true association. Second, in Kim et al.’s study, the authors recruited cases and controls from different source populations during an unknown period for controls. In this case, comparability issues can arise that could lead to bias of the association.

There are several strengths in our study. Case and control participants were recruited from one institution under the same protocol simultaneously. Therefore, comparability was secured. Additionally, interviewers were well trained for the whole survey procedure, biospecimens were handled under a tightly controlled protocol, and measurements for biomarkers were performed as one batch so that no experimental bias would affect the results.

There are several limitations in this study. First, samples were limited to a single institution, possibly compromising the representativeness of the data. However, the chance of selection bias was substantially reduced by recruiting control patients from the same department in the same hospital due to similar referral pattern for cancer and non-cancer patients to Division of Endocrinology in SMC. Healthy controls from the health screening center in the same hospital would have induced more selection bias because their catchment area was very different from the case population. Second, as mentioned before, the source population of the control group is people with benign thyroid diseases. If UIC was also associated with control diseases, the UIC levels of the case and control groups would have been similar to each other, leading the ORs toward the null. Third, there is a limitation in estimating the normal intake of iodine because a spot urine test was used. However, the limitation is non-differential between case and control groups leading the ORs toward the null. Therefore, the true association between creatinine-adjusted UIC and the risk of PTC might be greater than that observed in our study.

## Conclusions

Creatinine-adjusted UIC had a positive association with the risk of PTC, especially among those who were younger than 45 years for both men and women. The fact that South Korea is a country with an iodine-rich diet and has a unique parabolic age-related incidence curve matches well with the linear relationship between UIC and the risk of PTC among the younger age group. Further studies on long-term iodine intake and the risk of PTC, especially among younger populations and male populations, are warranted.

## Data Availability

The data sets used and analysed during the current study are available from the corresponding author on reasonable request.

## References

[1] GLOBOCAN. *International Agency for Research on Cancer*. http://globocan.iarc.fr/ (2020).

[2] Korea Central Cancer Registry. *2019 Annual Report of the Korea Central Cancer Registry*.

[3] Davies L, Welch HG (2006). Increasing incidence of thyroid cancer in the United States, 1973–2002. JAMA.

[4] Li M, Dal Maso L, Vaccarella S (2020). Global trends in thyroid cancer incidence and the impact of overdiagnosis. Lancet Diabetes Endocrinol..

[5] Gschwandtner E (2016). Increase of papillary thyroid microcarcinoma and a plea for restrictive treatment: A retrospective study of 1391 prospective documented patients. Surgery.

[6] Davies L, Welch HG (2014). Current thyroid cancer trends in the United States. JAMA Otolaryngol. Head Neck Surg..

[7] Ahn HY, Park YJ (2009). Incidence and clinical characteristics of thyroid cancer in Korea. Korean J. Med..

[8] Natioinal Cancer Information Center. https://www.cancer.go.kr/ (2021).

[9] Horn-Ross PL (2001). Iodine and thyroid cancer risk among women in a multiethnic population: The Bay Area Thyroid Cancer Study. Cancer Epidemiol. Prev. Biomark..

[10] Cléro É (2012). Dietary iodine and thyroid cancer risk in French Polynesia: A case–control study. Thyroid.

[11] Cao L-Z (2017). The relationship between iodine intake and the risk of thyroid cancer: A meta-analysis. Medicine.

[12] Michikawa T (2012). Seaweed consumption and the risk of thyroid cancer in women: The Japan public health center-based prospective study. Eur. J. Cancer Prev..

[13] Zimmermann MB, Galetti V (2015). Iodine intake as a risk factor for thyroid cancer: A comprehensive review of animal and human studies. Thyroid. Res..

[14] Wainwright P, Cook P (2019). The assessment of iodine status–populations, individuals and limitations. Ann. Clin. Biochem..

[15] Sun H (2020). Association between urinary iodine concentration and thyroid nodules in adults: A cross-sectional study in China. BioMed Res. Int..

[16] Cakir E, Eskioglu E, Aydin Y, Ozkan SK, Guler S (2011). Urine iodine excretion ın patients with euthyroid noduler disease. Ann. Saudi Med..

[17] Lee JH (2017). Relationship between iodine levels and papillary thyroid carcinoma: A systematic review and meta-analysis. Head Neck.

[18] Lee J-H (2018). Case–control study of papillary thyroid carcinoma on urinary and dietary iodine status in South Korea. World J. Surg..

[19] Kim HJ (2017). Strong association of relatively low and extremely excessive iodine intakes with thyroid cancer in an iodine-replete area. Eur. J. Nutr..

[20] Won Y-J (2009). Nationwide cancer incidence in Korea, 2003–2005. Cancer Res. Treat..

[21] Jung K-W (2021). Prediction of cancer incidence and mortality in Korea, 2021. Cancer Res. Treat..

[22] Sohn SY, Kim HJ, Jang HW, Kim SW, Chung JH (2012). Usefulness of measurement of serum iodine level to assess the appropriate low iodine diet preparation. J. Korean Thyroid Assoc..

[23] Vought R, London W, Lutwak L, Dublin T (1963). Reliability of estimates of serum inorganic iodine and daily fecal and urinary iodine excretion from single casual specimens. J. Clin. Endocrinol. Metab..

[24] Lee HS, Min H (2011). Iodine intake and tolerable upper intake level of iodine for Koreans. Korean J. Nutr..

[25] McLean RM (2014). Measuring population sodium intake: A review of methods. Nutrients.

[26] Ji C (2015). Systematic review of studies evaluating urinary iodine concentration as a predictor of 24-hour urinary iodine excretion for estimating population iodine intake. Rev. Panam. Salud Publ..

[27] Andersen S, Karmisholt J, Pedersen KM, Laurberg P (2008). Reliability of studies of iodine intake and recommendations for number of samples in groups and in individuals. Br. J. Nutr..

[28] Corvilain B, Van Sande J, Dumont JE (1988). Inhibition by iodide of iodide binding to proteins: the “Wolff-Chaikoff” effect is caused by inhibition of H2O2 generation. Biochem. Biophys. Res. Commun..

[29] Katagiri R, Yuan X, Kobayashi S, Sasaki S (2017). Effect of excess iodine intake on thyroid diseases in different populations: A systematic review and meta-analyses including observational studies. PLoS ONE.

[30] Kim K (2021). Association between iodine intake, thyroid function, and papillary thyroid cancer: A case-control study. Endocrinol. Metab..

[31] Wang Y (2021). Analysis of the correlation between high iodized salt intake and the risk of thyroid nodules: A large retrospective study. BMC Cancer.

[32] Lou X (2020). The effect of iodine status on the risk of thyroid nodules: A cross-sectional study in Zhejiang, China. Int. J. Endocrinol..

[33] Kim JY, Kim KR (2000). Dietary iodine intake and urinary iodine excretion in patients with thyroid diseases. Yonsei Med. J..

